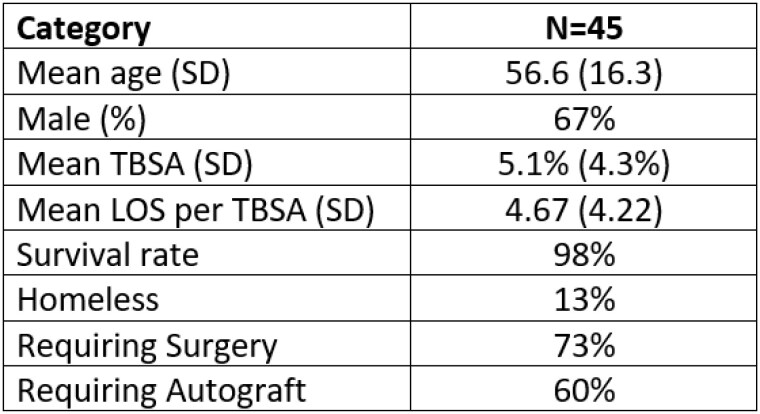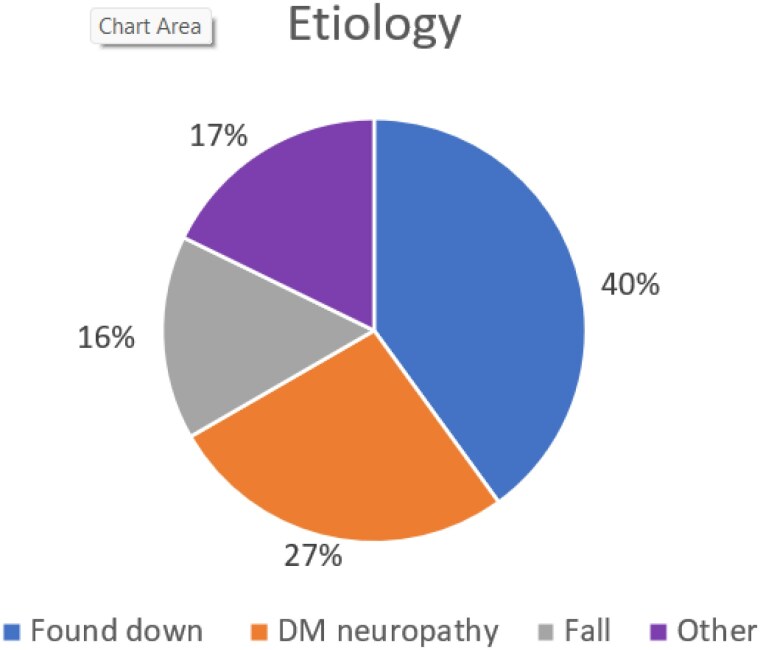# 642 A Retrospective Review of Pavement Burns Admitted to a Non-Desert Burn Center

**DOI:** 10.1093/jbcr/iraf019.271

**Published:** 2025-04-01

**Authors:** Syed Saquib, Sridharan Radhakrishnan

**Affiliations:** University of California Irvine Medical Center; University of California Irvine Medical Center

## Abstract

**Introduction:**

Hot contact pavement burns are an injury pattern traditionally seen in burn centers located in a desert climate. With the concern for worsening climate change, it is quite likely that burn centers not in a desert environment might have to tackle this problem as well.

**Methods:**

Using the burn registry, we performed a retrospective review of all hot pavement burns admitted to our ABA verified burn center from January 1, 2017 to July 30, 2024. We reviewed the following data points: age, gender, total body surface area (TBSA), length of stay (LOS), disposition, zip code of injury, etiologies, surgeries performed and comorbidities. For those pavement burns where a zip code of injury was identified, we used the Weather Underground database to determine the high ambient temperature on the day of injury.

**Results:**

We identified 45 pavement burns admitted to the hospital during this study period. 30 (67%) were males and had a mean age of 56.6 years (SD 16.3). 44 (98%) were over the age of 18. The mean TBSA was 5.1% (SD 4.3%). The mean time from injury to presentation to our hospital and subsequent admission was 4.8 days (SD 6.12). The most common etiologies were patients found down (18, 40%), patients with diabetic neuropathy (12, 27%), and falls (7, 16%). 6 patients (13%) were homeless. 13 (29%) of the burns occurred when the high ambient temperature on the day of injury in the zip code identified was over 100 degrees Fahrenheit. The mean high ambient temperature on the day of burn injury was 93.9 degrees Fahrenheit (SD 12.2). 33 (73%) patients required surgery. Of those 33 patients, 27 (82%) required a split thickness autograft. 44 (98%) patients survived their burns and were able to be discharged from the hospital. The mean LOS per TBSA was 4.67 days (SD 4.22).

**Conclusions:**

Pavement burn injuries are a unique injury pattern that often require hospitalization. While they typically do not involve a large TBSA, the overwhelming majority of these patients do require surgery. Most of these individuals needed a split thickness autograft to provide definite wound closure. This is a problem not unique to burn centers in a desert climate.

**Applicability of Research to Practice:**

As climate change continues to exacerbate extreme weather conditions, this injury pattern will likely be encountered with increasing frequency in many more burn centers. Therefore, it is incumbent upon on all burn centers to understand the presentation and management of this unique patient population.

**Funding for the Study:**

N/A